# “Why Aren’t You Listening?”: How Experiences of Endometriosis, Medical Dismissal, and Psychological Distress Should Influence Assessment and Interventions

**DOI:** 10.3390/healthcare14162496

**Published:** 2026-08-11

**Authors:** Panagiota Tragantzopoulou, Aikaterini Tragantzopoulou, Vaitsa Giannouli

**Affiliations:** 1 School of Social Sciences, University of Westminster, 115 New Cavendish St., London W1W 6UW, UK; g.tragantzopoulou@westminster.ac.uk; 2 School of Medicine, Aristotle University of Thessaloniki, 541 24 Thessaloniki, Greece; 3 Department of Psychology, Democritus University of Thrace, 691 00 Didymoteicho, Greece

**Keywords:** endometriosis, women’s health, chronic pain, mental health, psychological wellbeing, healthcare experiences, diagnostic delay

## Abstract

**Background/Objectives**: Endometriosis is a chronic gynecological condition affecting approximately 10% of women of reproductive age and is associated with chronic pain, fatigue, infertility, and reduced quality of life. Beyond its physical burden, delayed diagnosis, healthcare dismissal, and inadequate support may contribute substantially to psychological distress. In line with growing efforts to promote mental health and wellbeing within healthcare settings, this study explored women’s experiences of healthcare services and the psychological impact of living with endometriosis. **Methods**: A qualitative study was conducted using thematic analysis of 2500 publicly available Twitter/X posts shared by accounts self-presenting as women discussing experiences of endometriosis. **Results**: Three overarching themes were identified: (1) Institutional Invalidation of Women’s Endometriosis Experiences, (2) Living with the Multidimensional Burden of Endometriosis, and (3) Reimagining Endometriosis Care. Participants described chronic pain, reproductive uncertainty, and significant emotional distress that were frequently intensified by delayed diagnosis and experiences of not being believed or taken seriously within healthcare settings. Accounts suggested that suffering was shaped not only by disease symptoms but also by interactions with healthcare systems that undermined the legitimacy of women’s experiences. Conversely, validation, empathy, and collaborative communication were associated with improved wellbeing and engagement with care. Participants advocated for greater awareness of endometriosis, earlier diagnosis, and more integrated models of support. **Conclusions**: Endometriosis should be understood as a biopsychosocial condition whose impact extends beyond physical symptoms alone. The narratives analyzed highlight how psychological distress may be amplified by experiences of invalidation, delayed recognition, and fragmented care. Improving outcomes requires timely diagnosis, patient-centered healthcare interactions, and the integration of psychological support within multidisciplinary endometriosis services.

## 1. Introduction

Endometriosis is a chronic inflammatory gynecological condition characterized by the growth of endometrial-like tissue outside the uterine cavity [1,2]. Affecting approximately one in ten women and individuals assigned female at birth during their reproductive years, the condition is commonly associated with severe menstrual pain, chronic pelvic pain, painful sexual intercourse, fatigue, gastrointestinal symptoms, and reduced fertility [1,2]. Although various treatment options—including hormonal therapies, pain management strategies, and surgical interventions—can alleviate symptoms for some individuals, there is currently no definitive cure, and many patients continue to experience persistent physical and psychological burdens despite treatment [3,4].

Beyond its physical manifestations, endometriosis has profound implications for psychological wellbeing and everyday functioning. Persistent pain often disrupts education, employment, social participation, intimate relationships, and family life [2,5,6]. Many individuals report difficulties maintaining productivity, frequent absenteeism from work, and challenges performing routine daily activities [7]. The unpredictable nature of symptoms can create feelings of uncertainty and loss of control, contributing to heightened levels of emotional distress. Research consistently demonstrates elevated rates of anxiety, depression, stress, and reduced quality of life among individuals living with endometriosis [8,9], suggesting that the condition should be understood not solely as a gynecological disorder but also as a significant mental health concern.

One of the most challenging aspects of endometriosis is the prolonged pathway to diagnosis. Studies continue to report diagnostic journeys extending five to twelve years from symptom onset, often involving multiple healthcare consultations before referral to specialist services [2,10,11]. Individuals frequently describe a healthcare journey characterized by repeated consultations, ineffective treatments, and delayed or denied referrals. Qualitative research has shown that symptoms are often normalized, minimized, or attributed to other conditions, leading many patients to feel that their experiences are misunderstood or dismissed [2,12,13,14,15]. Such encounters can have important psychological consequences, including frustration, self-doubt, hopelessness, and reduced trust in healthcare systems [2,16,17]. In some cases, individuals begin questioning the legitimacy of their own symptoms after repeatedly encountering disbelief from healthcare professionals [2,16,17].

Emerging evidence suggests that interactions with healthcare providers play a critical role in shaping the emotional experiences of those living with endometriosis. Studies examining patient narratives have identified recurring reports of symptom trivialization, inadequate knowledge among healthcare professionals, misdiagnosis, and barriers to specialist referral [12,14,18]. These experiences often compel individuals to become active advocates for their own care, independently seeking information, conducting personal research, and negotiating access to specialist services [19]. While some patients eventually encounter compassionate practitioners who validate their concerns and facilitate appropriate treatment, many describe reaching a diagnosis only after years of persistence [16,17]. Consequently, the experience of endometriosis extends beyond physical symptoms and includes navigating healthcare systems that may inadvertently contribute to psychological distress.

Fertility-related concerns represent another significant dimension of the endometriosis experience. The condition is associated with an increased risk of infertility, which may generate considerable emotional burden for individuals who desire children [9,20,21]. Concerns regarding reproductive potential often coexist with feelings of uncertainty, grief, guilt, and fear about the future [2,5]. For some women, these concerns are intensified by social and cultural expectations surrounding motherhood and femininity, resulting in challenges to self-identity and personal wellbeing. The emotional impact of infertility and reproductive uncertainty further highlights the need to consider mental health outcomes when examining the broader consequences of endometriosis.

In response to unmet informational, emotional, and healthcare needs, many individuals turn to online communities and social media platforms [19]. Digital spaces have become important venues for sharing personal experiences, exchanging health-related information, seeking validation, and connecting with others facing similar challenges. Among these platforms, Twitter/X provides a unique environment in which users can publicly document their experiences, discuss treatment options, describe interactions with healthcare professionals, and advocate for greater awareness of endometriosis [22]. Social media narratives therefore offer valuable insights into the everyday realities of living with a chronic condition that is frequently misunderstood and underrecognized.

While previous research has explored clinical outcomes, diagnostic delays, and patient–provider interactions, fewer studies have examined how individuals living with endometriosis construct and communicate their experiences of endometriosis in online environments. Understanding these narratives is particularly relevant within the context of mental health and psychological wellbeing, as online discussions often reveal emotional needs, coping strategies, experiences of stigma, and perceptions of healthcare support. Examining these conversations can therefore provide important insights into how healthcare systems, clinical psychologists, and multidisciplinary professionals can better support individuals living with endometriosis.

The present study addresses this gap by examining how women construct meanings around healthcare encounters, recognition, and future expectations for endometriosis care through discussions on Twitter/X. Rather than focusing on the overall lived burden of endometriosis, this study specifically investigates how individuals describe interactions with healthcare systems, how they negotiate credibility and legitimacy in relation to their symptoms, and how they articulate expectations for more responsive models of care. By analyzing these narratives through a healthcare-focused lens, the study explores not only what challenges individuals experience, but also how those experiences are interpreted within broader processes of recognition, validation, and patient–provider relationships. This approach contributes to existing literature by examining patient-generated narratives as accounts of healthcare negotiation rather than solely as descriptions of illness burden. In doing so, the study contributes to ongoing discussions regarding patient-centered care and highlights the importance of integrating psychological perspectives into healthcare responses to chronic gynecological conditions.

## 2. Materials and Methods

### 2.1. Study Design

This study employed a qualitative research design to explore the experiences, perceptions, and expectations of individuals living with endometriosis as expressed through social media discussions. Twitter/X was selected as the data source because it provides a publicly accessible platform where people with endometriosis frequently share personal experiences, seek support, discuss healthcare encounters, and advocate for improved awareness and care. The study adopted a patient-centered perspective and used reflexive thematic analysis to identify patterns within online narratives concerning healthcare experiences and research priorities.

### 2.2. Data Collection

Publicly available posts were collected manually from Twitter/X. The first collection period took place between 1 September and 30 November 2023, and the second collection period took place between 1 February and 30 April 2024. Posts were collected on an ongoing basis throughout these periods rather than during a single retrieval session. The present dataset includes tweets analysed in our previous publication together with an additional collection period.

The additional February–April 2024 collection period was not intended to examine temporal changes in online discussions of endometriosis. Rather, these posts expanded the corpus available for reflexive thematic analysis by including additional healthcare-related narratives beyond the initial collection period. The second collection period included March, a month associated with international endometriosis awareness activities. Although this may have influenced the volume or visibility of online discussions during this period, the study did not aim to measure changes in posting frequency or public engagement over time. Instead, the additional posts were included to broaden the range of healthcare-related narratives available for qualitative interpretation. The potential influence of awareness-related activities is acknowledged as a contextual factor that may have shaped the online environment during data collection. Because the research question concerned the interpretation of healthcare experiences rather than changes over time, data from both periods were analysed together.

The current study represents a theoretically informed secondary qualitative analysis of this expanded corpus. While the previous study examined the broad lived experience of endometriosis, including symptom burden, quality of life, support seeking, and treatment experiences, the present analysis addresses a distinct research question focused on healthcare encounters, institutional recognition, and expectations for future care and research.

Data retrieval was conducted using Twitter/X’s native search function. No Twitter/X API, Academic Research API, automated scraping tools, or third-party data extraction software were used. Searches were performed manually using combinations of predefined endometriosis-related keywords, hashtags, and patient-community terms commonly used in online discussions. The search strategy was developed based on previous research examining online health communities and endometriosis-related social media discourse.

The primary search terms included: “endometriosis,” “#endometriosis,” “#endo,” “#endowarrior,” “#endocommunity,” and “#endosupport.” These terms were combined with additional experience-related keywords to identify patient-generated discussions, including terms related to symptoms, healthcare encounters, diagnosis, treatment, and daily living. Search combinations included variations of:

(“endometriosis” OR “#endometriosis” OR “#endo” OR “#endowarrior” OR “#endocommunity” OR “#endosupport”) AND (“pain” OR “diagnosis” OR “doctor” OR “healthcare” OR “treatment” OR “surgery” OR “symptoms” OR “living with”).

Search terms were adapted iteratively during data collection to identify relevant discussions while maintaining alignment with the study objectives.

All retrieved posts were manually screened according to predefined eligibility criteria. Eligible posts were written in English and included first-person accounts, experiences, opinions, or reflections relating to living with endometriosis, healthcare experiences, diagnosis, treatment, symptom management, or expectations regarding future research and care. Accounts were manually reviewed using publicly available profile information. Posts were included when the account self-presented as a woman (e.g., through explicit profile descriptions, linguistic self-identification, usernames, or profile photographs where available). Because these indicators reflect online self-presentation rather than verified demographic characteristics, gender identity could not be independently confirmed.

Posts were excluded if they originated from commercial accounts, healthcare organizations, advocacy campaigns, conference announcements, promotional material, or automated accounts (bots), or if they did not contain personal experiences of endometriosis. Duplicate posts containing identical content were removed during screening. Replies to other users were excluded because they were considered context-dependent interactions rather than independent narratives. Retweets were excluded unless they included additional original commentary from the user, in which case only the accompanying user-generated content was considered eligible.

Potential bot-generated content was identified manually based on indicators including repetitive automated posting patterns, absence of personal experience narratives, highly promotional content, and account characteristics inconsistent with individual user participation. Where multiple eligible posts originated from the same account, all eligible posts were retained because each post represented a distinct contribution to the dataset.

Following screening, a final corpus of 2500 eligible posts was assembled. The final dataset consisted of 2500 eligible posts rather than 2500 unique individuals. The number of unique accounts was not considered the primary unit of analysis because this study examined individual posts as patient-generated narratives rather than estimating the number of participants represented. Multiple posts from the same account were retained when they contained distinct relevant experiences, reflections, or discussions, as repeated contributions may represent ongoing illness narratives rather than duplicate observations. However, because social media users may contribute multiple posts and demographic information cannot be independently verified, the number of posts should not be interpreted as representing the number of individuals with endometriosis represented in the dataset.

The final corpus size was not determined through statistical sampling or saturation criteria. Instead, the corpus was established pragmatically through an iterative screening process that balanced the breadth of available patient-generated discussions with the feasibility of conducting detailed reflexive thematic analysis. The final dataset was considered sufficiently information-rich to capture diverse experiences while allowing close engagement with individual posts and development of interpretative themes.

Table 1 presents the identification, screening, exclusion, and inclusion process leading to the final dataset of 2500 posts.

### 2.3. Data Analysis

Data were analyzed using reflexive thematic analysis (RTA) following the six-phase approach developed by Braun and Clarke [23]. The analysis was conducted from an interpretivist perspective, recognizing that knowledge is generated through the interaction between participants’ accounts and researchers’ interpretative engagement with the data. Accordingly, themes were conceptualized as analytic constructions developed through an active and reflexive process rather than as objective patterns existing independently within the dataset. Reflexive thematic analysis was selected because it provides a flexible approach for exploring patterns of shared meaning while acknowledging the active role of researchers in producing and interpreting themes.

The analysis began with repeated reading of all tweets to facilitate familiarization with the dataset and to gain an overall understanding of participants’ experiences. During this phase, the first author recorded initial analytic observations and reflexive notes to document emerging ideas and critically examine how personal assumptions, disciplinary training, prior qualitative research experience, and previous research on endometriosis might influence data interpretation.

The first author conducted the coding inductively, generating initial codes directly from participants’ accounts rather than applying a pre-existing coding framework or theoretical model. Coding was understood as an interpretative process rather than a purely descriptive procedure. Codes were continuously reviewed, refined, and reorganized as familiarity with the dataset increased and the researchers developed deeper interpretations of the meanings expressed across the tweets.

Codes were subsequently examined for similarities, relationships, and conceptual significance and were organized into candidate themes. Theme development involved repeated movement between coded extracts, the complete dataset, and emerging interpretations to ensure that themes represented coherent patterns of shared meaning while maintaining clear conceptual boundaries. Some tweets contributed to more than one theme, reflecting the interconnected and complex nature of participants’ experiences.

The first author was responsible for the primary data analysis and development of the initial thematic structure. The co-authors contributed through reflexive discussions, critical engagement with emerging interpretations, and examination of alternative ways of understanding the data. These discussions did not aim to achieve consensus or measure coding reliability; rather, they supported reflexive dialogue and strengthened the interpretative depth of the analysis, consistent with Braun and Clarke’s reflexive approach [23].

No qualitative data analysis software was used. Data management, coding, and theme development were conducted manually through systematic organization of coded extracts, analytic notes, and reflexive memos.

The research team brought complementary disciplinary perspectives to the analysis. The first and third authors have backgrounds in psychology, previous experience conducting qualitative research, and prior research examining women’s experiences of endometriosis. The second author is an undergraduate medical student who contributed a clinical perspective during reflexive discussions. Throughout the analytic process, the researchers remained aware that their disciplinary backgrounds, previous knowledge of endometriosis, and professional interests could shape the interpretation of participants’ accounts. Rather than attempting to eliminate these influences, reflexivity was used to critically examine how these perspectives informed coding, theme development, and interpretation, consistent with the interpretivist orientation of reflexive thematic analysis.

Throughout the analysis, the research team maintained an audit trail documenting coding decisions, theme development, and methodological reflections. Reflexive memo-writing was used throughout the analytic process to encourage ongoing examination of emerging interpretations and the researchers’ assumptions. Regular meetings among the research team provided opportunities to critically reflect on how the researchers’ professional backgrounds and perspectives influenced the analytic process and the development of themes. These discussions aimed to enrich interpretation by incorporating multiple perspectives rather than establishing a single definitive account of participants’ experiences.

Throughout the analytic process, the authors repeatedly returned to the original dataset to ensure that the final themes remained grounded in the meanings expressed within participants’ accounts while acknowledging that all qualitative interpretations are shaped by the researchers’ analytic perspectives.

The final thematic structure consisted of three overarching themes that captured interpretative patterns regarding participants’ experiences of living with endometriosis, their encounters with healthcare systems, and their expectations for improved care and future research.

### 2.4. Ethical Considerations

This study analyzed publicly available content posted on Twitter/X and did not involve direct interaction with users or the collection of private information. Ethical approval was obtained from the relevant institutional research ethics committee prior to data collection.

Although the analyzed content was publicly accessible, the study was conducted in accordance with established ethical guidance for internet-mediated research and with Twitter/X Terms of Service applicable during the period of data collection. Particular consideration was given to the distinction between publicly available information and users’ reasonable expectations of privacy, as well as the potential risks associated with reporting online narratives.

No usernames, profile information, profile photographs, geographical identifiers, hyperlinks, or other potentially identifying information were collected, stored, or reported. The study did not retain searchable copies, screenshots, or archived versions of original posts. Only anonymized textual data required for analysis were maintained.

To further minimise the possibility of identification through online search engines, all examples presented in Section 3 are illustrative paraphrases rather than verbatim quotations. The paraphrases were carefully developed to preserve the original meaning, context, and intent of users’ posts while modifying wording sufficiently to reduce traceability. Quotation marks are therefore used only to indicate illustrative participant accounts and do not represent direct reproductions of original tweets.

The researchers adopted a non-participatory role throughout the study and did not interact with users, respond to posts, or engage with online communities during data collection or analysis.

## 3. Results

**Figure 1:**
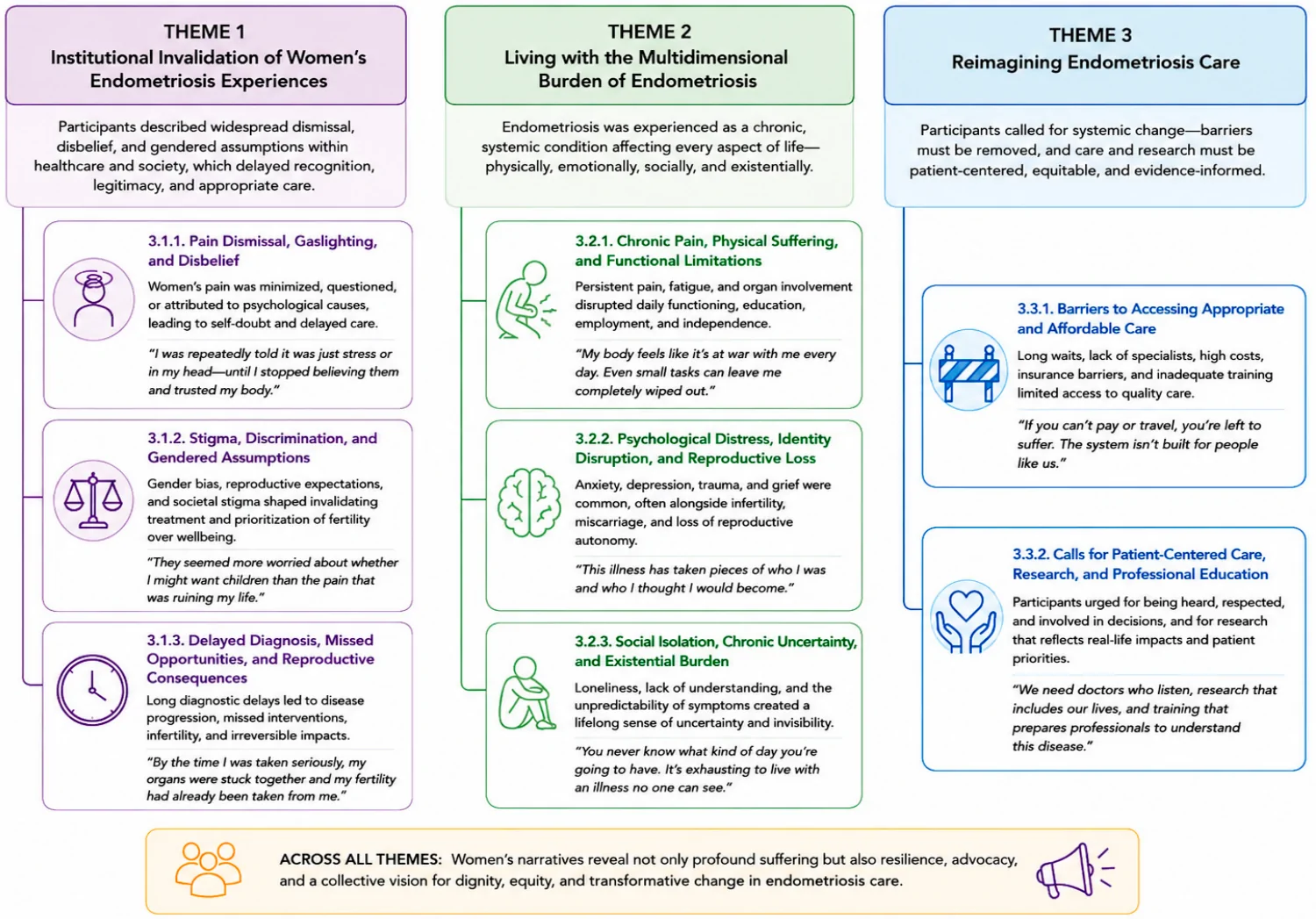
Thematic Framework of Women’s Experiences of Endometriosis from Social Media Narratives.

Analysis of the tweets revealed three overarching themes reflecting patients’ experiences of living with endometriosis and their expectations from healthcare systems and future research: (1) Institutional Invalidation of Women’s Endometriosis Experiences, (2) Living with the Multidimensional Burden of Endometriosis, and (3) Reimagining Endometriosis Care (Figure 1). These themes were analytically interconnected rather than independent categories. Together, they illustrate how experiences of endometriosis are shaped through the interaction between healthcare recognition, the ongoing physical and psychosocial burden of the condition, and patients’ efforts to advocate for more responsive and patient-centered models of care. It should be noted that all participant excerpts presented below are illustrative paraphrases of publicly available tweets. They are not reproduced verbatim in order to minimize the risk of identifying individual users while preserving the meaning and context of the original posts. Throughout the Section 3, the term ‘participants’ is used to refer to users whose posts met the inclusion criteria. Because gender identity and diagnosis could not be independently verified, references to women and individuals with endometriosis reflect how users self-presented within their posts or profiles rather than independently confirmed characteristics.

### 3.1. Institutional Invalidation of Women’s Endometriosis Experiences

The most pervasive interpretive pattern across the dataset concerned the ongoing negotiation of credibility within healthcare encounters. Participants’ accounts suggested that living with endometriosis involved not only managing symptoms but also repeatedly establishing the legitimacy of their own bodily knowledge. In this sense, institutional invalidation was experienced as more than delayed recognition of disease; it represented a process through which participants perceived their authority over their own experiences as being questioned. Users described navigating healthcare systems in which recognition was often achieved only after biomedical confirmation, positioning diagnosis as both a clinical outcome and a form of social and epistemic validation.

#### 3.1.1. Pain Dismissal, Gaslighting, and Disbelief

A dominant feature of participants’ accounts was the normalization and dismissal of severe symptoms. Participants described presenting with incapacitating pelvic pain, gastrointestinal symptoms, and other manifestations of endometriosis only to be reassured that these experiences reflected normal menstruation or psychological distress rather than underlying pathology. These accounts suggested that dismissal was rarely experienced as an isolated encounter but instead accumulated across repeated healthcare interactions.

Several participants reflected on early experiences in which their symptoms were reframed as behavioral or emotional exaggeration. One participant described repeated school absences due to pain being interpreted as “*overreacting and misusing sickness absence,*” despite later receiving an endometriosis diagnosis. Another explained that years of negative investigations led the doctors to believe they were “*imagining severe symptoms that I couldn’t else could explain.*”

Many participants explicitly used the language of medical gaslighting to describe their experiences. One user reflected that only after receiving a diagnosis did they realize the extent to which they had been repeatedly dismissed “*made me question my own reality*,” emphasizing that validation came not from clinical encounters but from finally receiving diagnostic confirmation through clinical evaluation, which they experienced as validating their previous symptoms. Participants described diagnostic confirmation as personally validating, often interpreting it as evidence that their previous concerns had been legitimate. One participant described diagnosis as emotionally transformative, explaining that it “*proved I had not been exaggerating or misinterpreting pain for years.*”

Importantly, several participants reported that invalidation continued even after diagnosis. Despite formal confirmation of endometriosis, some described ongoing scepticism regarding the severity of their pain, while others emphasized feeling ignored or interrupted during consultations. One participant summarized this experience by asking, “*Why aren’t you listening?*” Collectively, these accounts suggest that institutional invalidation functioned as a cumulative process that eroded confidence in one’s own bodily knowledge and contributed to emotional exhaustion and delayed help-seeking.

#### 3.1.2. Stigma, Discrimination, and Gendered Assumptions

Participants’ accounts also highlighted how experiences of invalidation were shaped by broader social and cultural narratives surrounding gender, pain, and reproduction. Many users interpreted their interactions with healthcare systems as embedded within a wider context in which female pain is normalized and undervalued.

A recurring comparison across narratives involved the perceived disparity between how endometriosis is treated and how conditions affecting men might be prioritized. One participant suggested that if a similar proportion of men experienced chronic pain, infertility, and organ dysfunction, “*it would be treated as a public health emergency rather than a condition to be managed individually.*” This sentiment reflected a broader perception that women’s health conditions are systematically deprioritized within research and clinical agendas.

Participants also described multiple forms of discriminatory assumptions within clinical encounters. Some reported that their symptoms were attributed to body weight or lifestyle factors rather than investigated medically, with one participant recounting that consultations repeatedly focused on “*diet and exercise explanations despite escalating pain and organ-related symptoms.*” Others described assumptions related to age or sexual activity, including instances where diagnostic procedures were questioned based on perceived sexual history rather than clinical need.

A particularly salient dimension of stigma concerned reproductive identity. Many participants perceived that healthcare professionals framed endometriosis primarily in relation to fertility preservation, often positioning potential future pregnancy as a therapeutic goal or justification for delaying intervention. One participant described being advised in consultations that pregnancy might alleviate symptoms, despite her primary concern being chronic pain management. Another reflected on feeling that her identity within healthcare settings was reduced to reproductive potential, stating that they were treated “*as though my future fertility mattered more than my current quality of life.*”

Participants who did not wish to have children often experienced these interactions as particularly invalidating, describing frustration at having their autonomy implicitly questioned. One user recalled a consultation in which their childlessness was met with visible pity, which they (i.e., the user) interpreted as an assumption that their life was somehow incomplete or diminished. Others who experienced infertility described a different but related form of distress, in which reproductive loss was acknowledged clinically but not supported emotionally.

Collectively, these accounts demonstrate how gendered expectations regarding motherhood, sexuality, and femininity shaped clinical interpretations of pain and treatment decisions. In this context, endometriosis care was frequently experienced not only as medically inadequate but also as socially structured by assumptions about what women should endure and prioritize.

#### 3.1.3. Delayed Diagnosis, Missed Opportunities, and Reproductive Consequences

A further key aspect of institutional invalidation was the extensive delay in obtaining diagnosis, with participants commonly reporting diagnostic journeys spanning many years, and in some cases decades. These delays were not described as neutral clinical outcomes but as consequences of repeated dismissal, inadequate investigation, and failure to recognize symptom patterns.

Participants frequently reflected on the contrast between long histories of severe symptoms and later clinical assessments that participants interpreted as indicating substantial disease burden. One user described learning after surgery that extensive internal adhesions had reportedly been identified after years of symptoms, despite previous investigations that the participant recalled as being interpreted by healthcare professionals as non-concerning. Another participant reported receiving a diagnosis only after a series of acute reproductive complications, describing this as “*a moment that retrospectively explained years of unaddressed pain*”.

Many participants interpreted delayed diagnosis as having contributed to worsening symptoms, increased suffering, or missed opportunities for earlier management. Narratives often included reflections on lost opportunities for earlier intervention, with participants suggesting that earlier recognition might have improved symptom management, supported quality of life, or altered their perceived reproductive trajectories. One participant reflected that they “*spent years being reassured instead of investigated*,” perceiving that by the time diagnosis occurred, the condition had already had lasting consequences for her health and life plans.

The emotional significance of these delays was also evident. Participants frequently described diagnostic confirmation as both validating and distressing, representing relief at finally being believed alongside grief for the years lost to untreated illness. Several expressed anger at the systemic failures that allowed prolonged suffering, framing delay not as incidental but as structurally produced. As such, delayed diagnosis functioned as a key mechanism through which institutional invalidation was enacted. Participants described these experiences as reinforcing feelings of disbelief, prolonged suffering, and significant emotional and physical burden over time.

### 3.2. Living with the Multidimensional Burden of Endometriosis

Participants’ narratives constructed endometriosis not simply as a collection of physical symptoms but as a disruptive experience that reshaped multiple dimensions of everyday life. The burden described across accounts extended beyond pain to include uncertainty, altered identities, disrupted life expectations, and continuous adaptation to an unpredictable condition. Endometriosis was therefore experienced as an ongoing negotiation between the body, personal identity, and social participation, where symptoms influenced not only functioning but also how participants understood themselves and their future possibilities.

#### 3.2.1. Chronic Pain, Physical Suffering, and Functional Limitations

Participants described chronic pain as the most immediate and pervasive dimension of living with endometriosis. Alongside persistent pelvic pain, they reported fatigue, gastrointestinal symptoms, bladder and bowel involvement, and complications associated with adhesions and repeated surgical interventions. Symptoms were commonly portrayed as continuous rather than confined to menstruation, requiring constant management and adaptation.

Many participants emphasized that pain affected every aspect of everyday life. One participant described “*living with pain most days that drained me physically and financially,*” while others highlighted the cumulative burden of ongoing treatment costs, repeated medical appointments, and medication use. Functional limitations were also common, with participants describing disrupted education, reduced work participation, and withdrawal from everyday activities. One participant reported being “*barely able to attend school at all,*” whereas another reflected that they had “*lost years of productivity due to flare-ups and recovery from surgeries.*”

Participants also described the invisibility of their symptoms as an additional burden. Because their condition was often not outwardly apparent, they frequently felt compelled to explain or justify their limitations to others. As one participant stated, “*People assume I am fine because they cannot see it, yet I am constantly managing pain that reshapes every part of my day.*” Another explained that the greatest burden was “*the constant need to justify why the pain makes normal life impossible.*” These accounts illustrate that the impact of chronic pain extended beyond physical suffering to include the ongoing work of legitimizing invisible illness in everyday social contexts.

#### 3.2.2. Psychological Distress, Identity Disruption, and Reproductive Loss

Alongside physical burden, participants described substantial psychological and emotional impact arising from living with endometriosis. Emotional distress was often framed as cumulative, resulting from prolonged pain, repeated medical encounters, uncertainty about prognosis, and ongoing invalidation. Many accounts reflected experiences of anxiety, depressive symptoms, emotional exhaustion, and loss of trust in healthcare systems. Participants frequently described the condition as destabilizing their sense of self. One user reflected that the disease had fundamentally altered their life trajectory, stating that they felt they had “*lost my sense of identity along with my physical stability and wellbeing.*” Others described emotional depletion resulting from prolonged attempts to seek diagnosis and treatment, with one participant summarizing this experience as feeling “*drained by years of having to fight simply to be taken seriously.*”

Medical trauma also emerged as a significant psychological dimension. Repeated dismissals, invasive investigations, and emergency interventions contributed to a sense of cumulative distress. Several participants described feeling emotionally worn down by repeated cycles of hope and disappointment, particularly when treatments failed to provide lasting relief. Reproductive loss and infertility were particularly powerful sources of emotional burden. Participants who experienced miscarriage or infertility often framed these experiences as deeply disruptive life events, compounded by the chronic nature of the disease. One participant described being informed of infertility after severe reproductive complications, reflecting that they had “*not only lost a pregnancy but also the possibility of future motherhood I had once assumed was available to me.*”

Others emphasized the psychological impact of reproductive uncertainty, including the distress of being advised that fertility might be compromised at a young age. One participant explained that being told their reproductive future was uncertain made them feel that they had “*lost the ability to make life decisions on my own terms.*” Across narratives, reproductive identity was frequently intertwined with broader questions of bodily autonomy and future planning. Participants often described feeling that endometriosis had interrupted or constrained expected life trajectories, including decisions about relationships, family planning, and personal aspirations.

#### 3.2.3. Social Isolation, Chronic Uncertainty, and Existential Burden

A further dimension of the burden related to the social consequences of living with a fluctuating and poorly understood chronic illness. Participants frequently described withdrawing from social activities due to pain, fatigue, or fear of symptom onset in public settings. This withdrawal often contributed to feelings of isolation and reduced participation in previously meaningful activities. Many participants also described the emotional strain of living with unpredictability. The inability to anticipate symptom severity from day to day created ongoing uncertainty, which shaped planning and social engagement. One participant reflected that they “*never knew which version of my health I would wake up to*,” making it difficult to commit to work, travel, or social arrangements.

This unpredictability was often linked to a broader sense of existential burden. Participants described feeling that life was placed on hold or constrained by the need to constantly adapt to symptoms and medical appointments. One user self-presenting as woman summarized this experience by stating that endometriosis had “*put large parts of my life on pause, as I was constantly managing pain, recovery, or waiting for treatment that never fully resolved the problem.*”

For some participants, the cumulative burden extended into expressions of emotional despair and severe psychological distress. A small number of accounts explicitly referenced suicidal ideation or feelings of hopelessness, often framed within the context of prolonged suffering and lack of effective treatment pathways. These reflections underscore the extent to which endometriosis was experienced not only as a physical illness but as a condition capable of profoundly affecting mental health and overall quality of life.

### 3.3. Reimagining Endometriosis Care

While participants’ narratives contained extensive accounts of suffering and healthcare failures, they also revealed an active process of reclaiming knowledge and defining what meaningful care should involve. Participants positioned themselves not only as recipients of medical services but as experts of their own embodied experiences, challenging healthcare models that prioritized biomedical outcomes while overlooking everyday realities. This theme therefore reflects not only demands for improved services but also a broader reconfiguration of whose knowledge is valued in shaping endometriosis care and research priorities.

#### 3.3.1. Barriers to Accessing Appropriate and Affordable Care

A dominant concern across narratives was the difficulty of accessing timely, specialist, and affordable care. Participants consistently described healthcare systems as slow, fragmented, and under-resourced, with long waiting times and limited availability of clinicians with expertise in endometriosis care. These structural barriers were often framed as directly contributing to disease progression and prolonged suffering.

Many participants described a sense of being forced into private healthcare systems as the only viable route to treatment, particularly when public services failed to provide timely intervention. One participant reflected that “*meaningful treatment was only accessible if I could afford to bypass the public system entirely,*” highlighting the emergence of financial capacity as a determinant of health outcomes. Others described escalating financial strain associated with this pathway, including the cumulative costs of consultations, diagnostics, and surgical interventions. In more extreme reflections, one participant noted that they had considered “*crowdfunding or drastic financial sacrifices in order to access surgery abroad or privately,*” illustrating the desperation produced by delayed care.

Insurance systems and administrative barriers were also frequently discussed as obstructive. Participants reported treatment denials, restricted coverage for specialist procedures, and inconsistent access to diagnostic imaging and pain management. Appointment cancellations and repeated placement on waiting lists were described as routine experiences, contributing to a perception of systemic instability and lack of continuity in care.

A further concern centered on workforce limitations and uneven clinical expertise. Participants frequently emphasized that general practitioners and non-specialist gynecologists often lacked sufficient training to recognize or manage endometriosis effectively. One participant described the experience of repeated consultations with clinicians who “*did not appear to understand the complexity of the condition or how urgently it required specialist input.*” This perceived gap in expertise was consistently linked to delays in diagnosis and suboptimal treatment pathways.

Collectively, these accounts reflect a perception that access to appropriate endometriosis care is not only medically constrained but also structurally stratified, with geography, funding, and specialist availability shaping outcomes as much as clinical need.

#### 3.3.2. Calls for Patient-Centered Care, Research, and Professional Education

Alongside critiques of access barriers, participants articulated a clear and consistent vision for improved healthcare delivery grounded in patient-centered principles. Central to this vision was the need for clinicians to listen to patients, validate their experiences, and engage in collaborative care relationships that recognize patients as credible experts in their own bodies.

Many participants described rare but significant positive encounters with healthcare professionals that contrasted sharply with their usual experiences of dismissal. One participant reflected on the emotional impact of finally being taken seriously, stating that it felt like “*the first time my concerns were treated as real rather than speculative.*” Another described a consultation in which a clinician’s acknowledgement of their symptoms was experienced as “*a moment of relief after years of not being believed.*” These accounts highlight that validation itself was experienced as a therapeutic intervention, underscoring the relational dimensions of care in chronic illness management.

Participants repeatedly called for improved professional education and training, particularly in relation to early symptom recognition, diagnostic pathways, and long-term management strategies. Many expressed the view that inadequate education among healthcare providers contributed directly to delayed diagnosis and inconsistent treatment. One participant suggested that “*better training in endometriosis could prevent years of unnecessary suffering and repeated misdiagnosis,*” positioning education as a primary site for systemic change.

Compassionate communication was also consistently emphasized as a core component of effective care. Participants described a desire for clinicians who not only understood the biomedical aspects of the condition but also acknowledged its emotional and social impact. Shared decision-making was frequently referenced as an ideal model of care, contrasting with experiences in which treatment decisions were perceived as paternalistic or narrowly focused on reproductive outcomes.

Research priorities were another key area of concern. Participants widely expressed frustration at perceived gaps in scientific understanding, particularly regarding disease etiology, non-surgical treatments, long-term management, and mental health impacts. Endometriosis was frequently described as under-researched relative to its prevalence and burden, reinforcing perceptions of systemic neglect in women’s health research.

In response, participants advocated for research agendas that extend beyond surgical outcomes and fertility metrics to include quality of life, chronic pain management, psychological wellbeing, and functional capacity. One participant emphasized the importance of shifting research focus toward “*how the condition actually affects everyday living, not just reproductive outcomes or surgical findings.*”

Notably, online patient communities were frequently described as alternative sources of knowledge and support. Participants often contrasted peer-generated information with clinical encounters, with one stating that “*other patients and online communities had provided more practical understanding of the condition than many healthcare professionals encountered in clinical settings.*” This reflects a redistribution of epistemic authority, where lived experience-based networks partially compensate for perceived institutional gaps.

## 4. Discussion

This study explored how users discussing endometriosis on Twitter/X constructed meanings around healthcare encounters, recognition, and expectations for future care. Three interrelated themes were identified: (1) institutional invalidation of endometriosis experiences, (2) living with the multidimensional burden of endometriosis, and (3) reimagining endometriosis care. Rather than describing the general burden of living with endometriosis, the present analysis focused on how participants interpreted interactions with healthcare systems and negotiated questions of credibility, legitimacy, and recognition. Collectively, the findings illustrate how participants constructed meanings around bodily suffering, healthcare recognition, and legitimacy within medical contexts. Importantly, the accounts analyzed suggest that distress associated with endometriosis was described not only in relation to symptoms themselves but also alongside experiences of uncertainty, inadequate support, and perceived challenges in having their experiences recognized within healthcare interactions.

A central finding concerns the patterns of institutional invalidation described within participants’ accounts. Participants’ narratives suggest that dismissal, normalization of pain, and psychological attribution of symptoms were experienced as recurring features of their healthcare encounters rather than isolated events. While previous research has documented the normalization of menstrual pain and diagnostic delays in endometriosis [12,13,15,24], the present findings extend this literature by illustrating how, within the narratives examined, repeated experiences of not being believed or adequately assessed were associated with declining trust in healthcare systems and uncertainty regarding embodied self-knowledge. Rather than reflecting only delayed diagnosis, these accounts suggest that participants experienced difficulties obtaining recognition throughout their healthcare journeys.

Within this context, participants’ narratives reflected what can be understood as epistemic injustice, whereby individuals are discredited as knowers of their own lived experience [25]. The frequent descriptions of being reassured that symptoms were “normal,” “exaggerated,” or “psychological” until later diagnostic confirmation illustrate how participants perceived a hierarchy between biomedical explanations and experiential knowledge. Although the term “medical gaslighting” is contested within clinical discourse [26], the accounts in this study point to an epistemic imbalance in which some participants experienced their suffering as being legitimized only after diagnostic confirmation. Importantly, these findings should not be interpreted as suggesting that symptoms require surgical or pathological confirmation before they are considered legitimate. Current guidance from the European Society of Human Reproduction and Embryology (ESHRE) emphasizes that endometriosis assessment should be based on symptom presentation, clinical evaluation, and appropriate investigations, with surgical confirmation no longer required in all cases [27]. Rather, the findings highlight how participants experienced diagnostic confirmation as personally meaningful because it represented recognition after prolonged experiences of dismissal. Clinically, this reinforces the importance of acknowledging and responding to patients’ reported symptoms throughout the diagnostic process, irrespective of whether definitive confirmation has yet been achieved.

These findings are consistent with previous qualitative interview studies exploring lived experiences of endometriosis, which have similarly identified themes of diagnostic uncertainty, frustration with healthcare encounters, and the importance of being believed by healthcare professionals [2,7]. However, the present study extends this literature by illustrating how these experiences are articulated within a naturally occurring online environment, where individuals publicly negotiate illness meanings, exchange knowledge, and seek validation from others. Unlike our previous analysis [19], which examined dismissal and diagnostic delay as part of the broader lived burden of endometriosis, the present study interprets these experiences through the lens of healthcare recognition, credibility, and the relationship between patients’ embodied knowledge and biomedical systems. At the same time, the nature of social media data requires careful interpretation, as individuals who actively share their experiences online may differ from those who do not engage in digital health communities. Therefore, the findings should be understood as complementary to, rather than representative of, the broader population of people living with endometriosis.

These dynamics are embedded within broader gendered structures of healthcare. Participants’ accounts suggest that experiences of dismissal and delay may reflect historically described patterns in which women’s pain is more likely to be normalized, minimized, or interpreted through psychological frameworks. Consistent with prior literature [16,24], participants frequently described symptoms being interpreted through psychological or behavioral explanations rather than investigated as possible indicators of gynecological pathology. Notably, some participants perceived that clinical attention was disproportionately directed towards fertility preservation rather than pain relief, which they interpreted as reinforcing the prioritization of reproductive outcomes over broader health concerns. This reflects broader critiques of biomedical systems in which women’s bodies are positioned primarily in relation to reproduction, rather than as sites of autonomous health experience [19,28].

Diagnostic delay and psychological distress emerged as closely interconnected within participants’ accounts. While previous studies have documented delays in diagnosis of up to a decade [2,10], the present findings emphasize how participants interpreted these delays within their own illness narratives. Delay was described not as a neutral clinical interval but as a period characterized by repeated uncertainty, frustration, perceived dismissal, and emotional distress. Diagnosis, when finally achieved, was therefore experienced ambivalently: it provided validation of suffering while simultaneously producing anger, grief, and regret over years of unrecognized illness. Participants also described anxiety, exhaustion, identity disruption, and hopelessness that were linked not only to pain but also to the ongoing effort required to obtain recognition, support, and appropriate care. While elevated levels of depression and anxiety have been previously documented in endometriosis populations [5,6], the present findings suggest that psychological distress was understood by participants as emerging through the interaction between embodied illness experiences and perceived healthcare invalidation, rather than from symptom burden alone.

The social consequences of endometriosis further highlight the visibility paradox of chronic illness [29,30]. Participants described withdrawing from social participation while simultaneously feeling compelled to explain or justify their condition to others. These experiences illustrate how living with an often invisible condition may contribute to isolation and uncertainty regarding whether suffering will be recognized by others [31].

The theme of reimagining endometriosis care highlights participants’ expectations for fundamentally different models of healthcare engagement. Across accounts, validation emerged as a central therapeutic mechanism. Being listened to, believed, and taken seriously was frequently described as transformative, regardless of immediate clinical outcomes. This underscores the importance of relational aspects of care, suggesting that clinical encounters are not solely sites of diagnosis and treatment but also of recognition and epistemic affirmation. Such findings align with person-centered care frameworks [32] while highlighting the importance of integrating patients’ experiential knowledge within evidence-based clinical practice.

Taken together, the findings position endometriosis as a complex biopsychosocial condition shaped not only by physiological pathology but also by epistemic, institutional, and gendered structures that influence whose knowledge is recognized in healthcare contexts. The distress associated with endometriosis within the narratives analyzed appears to involve more than symptom burden alone and was frequently described alongside experiences of uncertainty, dismissal, and inadequate support. Addressing these challenges requires not only improvements in diagnostic pathways and access to care, but also healthcare practices that recognize patients’ experiential knowledge, support shared decision-making, and integrate psychological and multidisciplinary care alongside biomedical management. Such approaches may contribute to reducing the clinical and psychosocial burden associated with endometriosis and improving the alignment between healthcare practice and patient needs.

### 4.1. Strengths and Limitations

This study contributes to the growing literature on endometriosis by examining women’s experiences through naturally occurring discussions on Twitter/X. Unlike interview-based studies, social media data provide access to spontaneous accounts generated outside formal research settings, allowing participants to describe their experiences in their own words and on their own terms. The large dataset also enabled the identification of recurring patterns across diverse experiences, capturing both individual narratives and broader collective concerns. Furthermore, the study extends existing research by highlighting the interconnected physical, psychological, social, and healthcare-related dimensions of endometriosis, emphasizing the relevance of patient-generated narratives for understanding lived experiences of chronic illness.

Several limitations should be acknowledged. First, the study relied exclusively on publicly available English-language tweets, which may not reflect the experiences of individuals who do not use social media, communicate in other languages, or choose not to discuss their health publicly. As with all social media research, demographic information such as age, ethnicity, socioeconomic status, educational level, and disease severity could not be independently verified, limiting the ability to contextualize experiences across different population groups.

Second, Twitter/X users may represent individuals who are particularly motivated to share experiences, seek support, or advocate for change, potentially amplifying accounts of both positive and negative healthcare experiences. This selection bias may influence the types of narratives captured, as individuals experiencing greater distress, dissatisfaction with healthcare, or stronger motivations for advocacy may be more likely to participate in online discussions. Conversely, individuals with limited digital access, lower health literacy, or less engagement with online communities may be underrepresented. Consequently, the findings should be understood as reflecting the perspectives of individuals who actively shared their experiences on Twitter/X rather than the full spectrum of experiences among people living with endometriosis.

Third, the character limitations inherent to the platform may restrict the depth and contextual detail available within individual posts. Fourth, because the study analysed publicly shared narratives, it was not possible to independently verify users’ reported diagnoses, treatments, or healthcare experiences. Similarly, although the screening process included manual review of publicly available profile information to identify accounts that self-presented as women, neither gender identity nor a diagnosis of endometriosis could be independently confirmed. Furthermore, multiple eligible posts may have originated from the same account; therefore, the number of tweets analysed should not be interpreted as representing the number of independent individuals. The findings should therefore be interpreted as reflecting self-presented accounts shared on Twitter/X rather than clinically or demographically verified characteristics.

Finally, healthcare experiences should be interpreted within their broader sociocultural and healthcare contexts. Social media narratives are shaped by differences in healthcare systems, referral pathways, availability of specialist services, healthcare financing, cultural beliefs regarding pain and reproductive health, and broader socioeconomic conditions [33,34,35,36]. These contextual factors may influence how endometriosis is experienced, interpreted, and discussed online. Future research combining social media analyses with interview-based approaches across diverse healthcare settings may provide a more comprehensive understanding of how these contextual factors shape illness experiences. Accordingly, the findings should be interpreted as reflecting the experiences represented within this dataset rather than as universally applicable across all healthcare settings or populations.

Despite these limitations, social media platforms remain valuable sources of insight into patient experiences, particularly for conditions such as endometriosis where individuals often encounter stigma, dismissal, and barriers to care [19]. The findings therefore provide an important perspective on how women publicly construct, negotiate, and make sense of living with endometriosis within contemporary healthcare systems.

### 4.2. Implications for Clinical Practice, Research, and Policy

The findings have important implications for healthcare professionals, clinical psychologists, researchers, and policymakers. First, the frequent accounts of symptom dismissal and diagnostic delay within this dataset highlight the importance of continued efforts to improve professional education regarding the recognition, assessment, and management of endometriosis. Earlier identification of symptoms and clearer referral pathways may help address delays in care, although future research is needed to evaluate these approaches across different healthcare contexts.

Second, the findings underscore the importance of adopting patient-centred and trauma-informed approaches to care, rather than stigmatising patients [36]. Participants frequently described validation and being believed as transformative aspects of positive healthcare encounters. Healthcare professionals should therefore recognize that listening to patients, acknowledging their experiences, and engaging in shared decision-making are not merely communication skills but essential components of effective clinical care.

Third, the substantial psychological burden described across narratives suggests that endometriosis management should extend beyond symptom control and reproductive outcomes. Integrated multidisciplinary services that include psychological assessment and support may help address anxiety, depression, grief, identity disruption, reproductive concerns, and the emotional consequences of chronic pain. Clinical psychologists may play an important role in supporting adjustment to illness, coping with uncertainty, managing chronic pain, and addressing the psychological effects of medical invalidation and reproductive loss. All the above must be incorporated in future assessment and intervention protocols which will also take into account the different cultural and medical contexts in which the patients live.

The findings also have implications for future research. Participants consistently called for research that reflects the realities of everyday life with endometriosis. Future studies should therefore move beyond traditional biomedical outcomes and incorporate quality of life, psychological wellbeing, social participation, occupational functioning, and patient-defined priorities. Greater attention should also be given to understanding the long-term psychological consequences of diagnostic delay and healthcare invalidation, as well as the potential role of online communities as sources of support, advocacy, and health information.

At a policy level, the findings reinforce growing calls for increased investment in endometriosis services, professional training, and research funding. Addressing workforce shortages, improving access to specialist care, and reducing financial barriers to treatment are essential steps toward reducing inequities in endometriosis care and improving patient outcomes. Future clinical pathways should consider how these findings may be translated into more integrated models of endometriosis care. This may include the development of clearer referral pathways for individuals presenting with persistent pelvic pain or related symptoms, improved coordination between primary care providers, gynaecologists, pain specialists, and mental health professionals, and earlier incorporation of psychological assessment and support. Such approaches should not replace biomedical investigation but rather complement it by addressing the emotional, social, and functional consequences of living with endometriosis. Future research should evaluate the effectiveness of multidisciplinary care models that combine timely diagnosis with psychological and supportive interventions tailored to individual needs and healthcare contexts.

## 5. Conclusions

This study highlights how women discussing endometriosis on Twitter/X constructed healthcare experiences not only as encounters with symptoms and treatment, but also as encounters with recognition, credibility, and validation. The findings suggest that, within these narratives, distress was frequently connected to the interaction between embodied illness experiences and perceived difficulties obtaining acknowledgement within healthcare systems.

The findings underscore the importance of recognizing endometriosis as a biopsychosocial condition that requires more than symptom-focused management. Improving care will require earlier diagnosis, greater validation of patients’ experiences, and the integration of psychological support within multidisciplinary treatment pathways. Healthcare professionals must move beyond viewing endometriosis solely as a gynecological disorder and acknowledge its broader impact on wellbeing, identity, and quality of life.

Ultimately, addressing endometriosis effectively requires healthcare systems that recognize women as credible experts on their own experiences and deliver genuinely patient-centered care. Such changes are essential for reducing the substantial psychological and social burden associated with the condition and improving outcomes for those affected.

## Figures and Tables

**Table 1:** Selection Process and Inclusion of Twitter/X Posts in the Final Qualitative Analysis Dataset.

Stage of Selection	Number of Posts	Description
Posts identified through Twitter/X searches	6800	Publicly available posts retrieved using predefined endometriosis-related keywords and hashtags during September–November 2023 and February–April 2024.
Posts screened for eligibility	6800	All retrieved posts were manually screened according to predefined inclusion and exclusion criteria.
Posts excluded during screening	4300	Posts excluded because they were promotional/commercial content, healthcare provider advertisements, conference-related announcements, advocacy campaigns without personal experiences, automated/spam accounts, duplicate content, replies, or unrelated to lived experiences of endometriosis.
Posts meeting eligibility criteria	2500	Posts containing first-person experiences, reflections, or discussions related to symptoms, diagnosis, treatment, healthcare encounters, support, or expectations for future care.
Final dataset included in thematic analysis	2500	Final corpus analyzed using reflexive thematic analysis.

Note: The final corpus comprised 2500 eligible posts, including 2000 posts from the previous publication (September–November 2023) and 500 additional posts collected during February–April 2024. These posts were combined and analyzed as a single qualitative dataset because the present study addressed a different research question rather than comparing temporal differences between collection periods.

## Data Availability

The data presented in this study are available on request from the corresponding author. The data are not publicly available due to ethical restrictions.
